# IL‐1β/IL‐1R1 signaling induced by intranasal lipopolysaccharide infusion regulates alpha‐Synuclein pathology in the olfactory bulb, substantia nigra and striatum

**DOI:** 10.1111/bpa.12886

**Published:** 2020-08-04

**Authors:** Haichen Niu, Qian Wang, Weiguang Zhao, Jianxin Liu, Deguang Wang, Bilal Muhammad, Xiaoyu Liu, Ning Quan, Haoyu Zhang, Fang Zhang, Yong Wang, Haiying Li, Rongli Yang

**Affiliations:** ^1^ Jiangsu Key Laboratory of Brain Disease and Bioinformation Xuzhou Medical University Xuzhou 221004 China; ^2^ Department of Genetics Xuzhou Medical University Xuzhou 221004 China; ^3^ Graduate School Xuzhou Medical University Xuzhou 221004 China; ^4^ Department of Geriatric Medicine Affiliated Hospital of Xuzhou Medical University Xuzhou 221004 China; ^5^ Department of Clinical Medicine Xuzhou Medical University Xuzhou 221004 China; ^6^ Department of human anatomy Xuzhou Medical University Xuzhou 221004 China; ^7^ Department of Neurology Affiliated Hospital of Xuzhou Medical University Xuzhou 221004 China; ^8^ Department of Biomedical Science Charles E. Schmidt College of Medicine and Brain Institute Florida Atlantic University Jupiter FL 33458 USA; ^9^ School of Marine Sciences Nanjing University of Information Science and Technology Nanjing 210044 China; ^10^ Laboratory of Morphology Xuzhou Medical University Xuzhou 221004 China; ^11^ Department of Neurology First Affiliated Hospital of Soochow University Suzhou 215006 China; ^12^ Department of Pathology Xuzhou Medical University Xuzhou 221004 China; ^13^ Department of Geriatrics Xuzhou Medical University Xuzhou 221004 China

**Keywords:** autophagy, IL‐1β/IL‐1R1, microglia, Parkinson’s disease, α‐Syn

## Abstract

Olfactory dysfunction is one of the early symptoms seen in Parkinson’s disease (PD). However, the mechanisms underlying olfactory pathology that impacts PD disease progression and post‐mortem appearance of alpha‐Synuclein (α‐Syn) inclusions in and beyond olfactory bulb in PD remain unclear. It has been suggested that environmental toxins inhaled through the nose can induce inflammation in the olfactory bulb (OB), where Lewy body (LB) is the first to be found, and then, spread to related brain regions. We hypothesize that OB inflammation triggers local α‐Syn pathology and promotes its spreading to cause PD. In this study, we evaluated this hypothesis by intranasal infusion of lipopolysaccharides (LPS) to induce OB inflammation in mice and examined cytokines expression and PD‐like pathology. We found intranasal LPS‐induced microglia activation, inflammatory cytokine expression and α‐Syn overexpression and aggregation in the OB via interleukin‐1β (IL‐1β)/IL‐1 receptor type I (IL‐1R1) dependent signaling. In addition, an aberrant form of α‐Syn, the phosphorylated serine 129 α‐Syn (pS129 α‐Syn), was found in the OB, substantia nigra (SN) and striatum 6 weeks after the LPS treatment. Moreover, 6 weeks after the LPS treatment, mice showed reduced SN tyrosine hydroxylase, decreased striatal dopaminergic metabolites and PD‐like behaviors. These changes were blunted in IL‐1R1 deficient mice. Further studies found the LPS treatment inhibited IL‐1R1‐dependent autophagy in the OB. These results suggest that IL‐1β/IL‐1R1 signaling in OB play a vital role in the induction and propagation of aberrant α‐Syn, which may ultimately trigger PD pathology.

## Introduction

Parkinson’s disease (PD) is characterized by progressive motor and non‐motor symptoms linked to alpha‐Synuclein (α‐Syn) pathology and the loss of dopaminergic neurons in the nigrostriatal system (11). Clinical research has found most PD patients have olfactory deficits and accumulation of intraneuronal Lewy bodies/Lewy neurites (LBs/LNs) containing misfolded fibrillar α‐Syn in the olfactory bulb (OB) prior to the onset of motor symptoms (32). However, the relationship linking olfactory dysfunction and PD development remains unclear.

Alpha‐Synuclein is a small protein of 140 amino acids that is specifically enriched in presynaptic nerve terminals and makes up about 1% of the total protein content in each neuron (34). Autosomal dominant *α‐Synuclein* gene mutations or gene amplification (duplication/triplication) have been implicated in early‐onset familial PD (13). This finding and additional evidence has strongly suggested α‐Syn plays an important role in the pathogenesis of PD (13). Initially, genetic and biochemical data suggested that overexpression of α‐Syn results in the formation of α‐Syn oligomers and PD pathology (3). Post‐mortem examinations have found three forms of α‐Syn in PD brains: monomeric, oligomeric and fibril (10). Under normal physiological conditions, naively folded monomeric α‐Syn is soluble, nontoxic and capable of binding to a variety of cellular membranes. Under pathological conditions, misfolded α‐Syn self‐associates into small oligomeric species, and then, into high‐molecular weight insoluble fibrils that are known to be toxic, not only on their own, but also propagate their aggregating property to nearby monomeric species between cells to form additional oligomeric species, acting as seeds to the generation of fibril aggregations (16, 46). Moreover, the α‐Syn oligomeric species capable of cell‐to‐cell transmission (pathologic α‐Syn) have been found to cause Parkinson’s‐like Lewy Body pathology in cells and mice (28). In familiar PD pathology more oligomers species of α‐Syn were found with the presence of Lewy bodies or Lewy neurites (20). As such, soluble α‐Syn oligomers have been proposed as key etiological molecules in the development of nigrostriatal dopaminergic cell vulnerability in PD (32, 47).

The sporadic form of PD accounts for the vast majority of clinical PD cases and it is not dependent on mutated α‐Syn. Therefore, it is important to identify factors that increase the expression of wild‐type α‐Syn and the transformation of α‐Syn from monomers to oligomers/fibrils in order to understand the development of sporadic PD. Notably, more than 90% of sporadic PD patients have significantly impaired olfactory function. As such odor identification tests have been implemented to help diagnose PD (24, 43). One should keep in mind, however, several distinct components of olfaction including odor detection, odor discrimination, odor memory and odor identification, will become gradually impaired during the normal aging process without PD (12, 59). Interestingly, overexpression of α‐Syn‐GFP fusion proteins in the OB has been shown to induce prodromal PD‐like changes in multiple rat brain regions 3 months after the α‐Syn overexpression (41). This evidence supports Braak’s hypothesis that PD begins in the structures of the lower brainstem and the olfactory system (6). Taken together, current evidence suggests the olfactory region could be where PD is initiated.

Anatomical studies have found that the olfactory sensory neurons (OSNs) located in the epithelium (OE) of the nasal cavity detect odor information in the environment and transmit it to the brain via the olfactory nerves (58). The OSNs is thus directly exposed to different external environmental toxins, making them readily insultable and the connected OB susceptible to inflammation. Moreover, the olfactory system is a highly evolved and complex neural network that communicates with different brain regions (57). This system can receive direct projections from the SN to the OB and send information to the SN by multisynaptic transmission (19). Our previous studies reported that a mutant α‐Syn in OB can be transferred to the SN to induce aggregative LBs in the SN where the pathological α‐Syn induces the loss of dopaminergic cells, leading to PD‐like motor symptoms in rats (43). Therefore, external agents might trigger inflammation of the OE and OB, which in term drives neurological dysfunction beyond OB.

Microglia are the resident immune cells of the CNS located throughout the CNS. They are derived from myeloid precursors in the embryonic yolk sac and account for 10%–15% of all cells types found in the adult brain (25). In physiological conditions, microglia has been described as “quiescent” or remaining in a “resting” state with a ramified shape, but they play a vital and complex role in neural physiology and in the progression of PD (8). These cells scan their environment to maintain homeostasis of the internal environment of the CNS. When facing attacks from different stimuli, microglia transform into a reactive phenotype with enlarged soma and release pro‐inflammatory factors to act as “danger” signals (8). Microglia in this state are termed activated microglia. Activated microglia become potent immune effector cells and initiate both innate and adaptive immune responses through different immuno‐signaling mechanisms (54). Interestingly, activation of microglia have been shown to produce and secrete Interleukin‐1β (IL‐1β) and to elevate α‐Syn expression (2).

Minocycline (Mino) is a semisynthetic tetracycline antibiotic and easily crosses the blood–brain barrier (49). Yrjänheikki J and his colleagues found that Mino could inhibit microglia activation in models of forebrain ischemia and PD (61). Mino treatment was reported to prevent the induction of caspase 1 and inducible nitric oxide synthase (52) from activated M1 microglia without affecting the expression of M2 microglia markers (26). In the present study, Mino is used to inhibit microglial activation and determine the involvement of microglia in LPS‐induced α‐Syn pathology in OB.

To test our hypothesis, the following experiments were performed to determine: 1) The effects of intranasal LPS infusion on the expression of α‐Syn and α‐Syn pathology in the OB; 2) The effects of inhibiting microglia on the changes induced by the LPS treatment; 3) The role IL‐1R1 and the site‐specific cellular location of IL‐1R1 signaling; and 4) the impact of the LPS treatment on the nigral‐striatal dopaminergic system.

## Materials and Methods

### Animals

C57BL mice (6–8 weeks old) were purchased from the Experimental Animal Center of Xuzhou Medical University. Breeders for IL‐1R1‐deficient (IL‐1R1^r/r^) mice and IL‐1R1 reporter (IL‐1R1^GR/GR^) mice were obtained from Dr. Ning Quan at Florida Atlantic University (Jupiter, USA). The breeding and genotyping of these transgenic mice were done in the home lab. Age‐ and sex‐matched littermate of the transgenic animals were used as controls. All animals were housed in groups under 12 h light/dark cycles with free access to food and water. All animals were handled in strict accordance with good animal practices and all procedures were completed under the specifications set by the Xuzhou Medical University Animal Care and Use Committee (protocol number: 2019004121M).

### Reagents and treatment

Lipopolysaccharides (LPS) was purchased from Sigma‐Aldrich (#L2880, St. Louis, MO, USA,) and diluted to 1 g/L for intranasal infusion in mice. Mice were anesthetized with 2% of isoflurane in 100% of O_2_ (at 1 L/min) via a tube connected to a head holder. Once anesthetized, mice were given a 10 μL of LPS intranasal administration to each nostril. The LPS was given once a day and mice were killed at different time points based on experimental designs.

Minocycline hydrochloride (MINO) was dissolved and administered in drinking water at a dose of 50 mg/kg per day for 3 days before the LPS administration (37). MINO was purchased from Sigma‐Aldrich (Shanghai, China, #M9511).

### Behavioral tests

Locomotor ability, anxiety, olfactory discrimination and motor function were examined by different behavior tests. All behavioral tests were done 6 weeks after the LPS treatment and were carried out between 9:00 and 15:00.

### Locomotor activity behavioral test

Locomotor activity was evaluated using an open‐field test as previously reported (27). Before the test, all mice were habituated in the open‐field apparatus once a day for three consecutive days for 1 hour. Then, mice in the open‐field were monitored by the infrared camera positioned above the apparatus connected to a computer. The videos were analyzed later with the ANY Maze video tracking system (Stoelting Co., Wood Dale, IL, USA).

### Elevated plus maze test

The elevated plus maze test was conducted as previously described to assess anxiety‐like behaviors (57). The elevated plus maze consists of two open arms with 3‐cm tall ledges and two closed arms (25 × 5 cm) with 15‐cm tall transparent walls. White plastic plates were used to build the floors of the arms and the central square (5 × 5 cm) at a height of 55 cm above the laboratory floor. The arms of the same type were arranged on opposite side of the maze. A white light (40 w) was placed on the ceiling to illuminate the maze. Each mouse was placed into the center of the maze facing one of the closed arms and its behavior was recorded for 10 minutes. The percentage of time spent in the open arms was measured using the Image EP software.

### Pole Test

The degree of bradykinesia was measured by the pole test (5). Briefly, the mouse was placed head‐upward on the top of a vertical rough‐surfaced pole (diameter 10 mm; height 50 cm) and the time until it descended to the floor (total time) was recorded with a maximum duration of 120 s. If the animal fell the rest of the way, the time when it reached to the floor was recorded. If the animal could not turn downward and instead dropped from the pole, the total time was recorded as 120 sec (default value). Animals were trained three times every day for 3 days before test. During the test, every animal was tested for three times and the average value was used. The rater was blinded to the experimental designations of the animal.

### Olfactory function test

Olfactory function was examined using the olfactory discriminating test as in previous studies (41). This test was done in the home cage to reduce the environmental stress. Lemon or mint was dissolved in the mineral oil as odor stimulus and mineral oil was used as vehicle. Different odors were presented by the cotton swab dropped the odors (10 uL every time). The whole experimental procedure included: (i) Habituation: mice were habituated to one odorant (lemon) through successive presentations (5 minutes). (ii) Dishabituation: after habituation, the mice were presented with a second novel odorant (mint) during a single test trial (5 minutes). The number of investigating sniffs to the second odorant was quantified, which was used as an index of odor discrimination between the two odor stimuli. If animals could detect the perceptual difference between the two odorants, they will spend more time sniffing the novel odor during the test. Conversely, if there were no perceptual difference between the two odors; the number of sniff behavior on the novel stimulus would be reduced.

### Isolation and dissection of the brain tissues

Animals (six mice per group) were sacrificed by cervical dislocation 6 weeks after the LPS administration. The OB, SN and striatum tissues of the brain were removed in ice‐cold conditions and stored at −80°C until further use. The methods for brain dissection have been described previously (38). The OB, SN and striatum tissues were then used for Western blot. Striatum tissue was also used to measure levels of dopamine and its metabolites via High‐Performance Liquid Chromatography (HPLC). Brain sections were homogenized in 500 μL of 0.1 M TCA (10–2 M sodium acetate, 10–4 M EDTA, 10.5% methanol) as previously described (43). Samples were centrifuged at 10 000 × *g* for 30 minutes to remove the supernatant. Catecholamine levels were analyzed by HPLC coupled with electrochemical detection with an Antec Decade II (Zoeterwoude, the Netherlands). Supernatant samples were injected with a water autosampler onto a Phenomenex Nucleosil (5u, 100A) C18 HPLC column (150 × 4.60 mm). Then, samples were eluted in a mobile phase followed by delivery of the solvent at 0.38 mL/min with a Waters 515 HPLC pump. The mobile phase composition was: 75.2 mM of sodium phosphate (monobasic, monohydrate), 1.39 mM of 1‐octanesulfonic acid (sodium salt, anhydrous), 0.125 mM of ethylene diamine tetra‐acetic acid, 0.0025% of triethylamine and 10% of acetonitrile; pH 3.0 adjusted with 85% of phosphoric acid. The levels of 3,4‐dihydroxyphenylacetic acid (DOPAC) and dopamine (DA) were detected. Waters Empower software was used for HPLC control and data acquisition. Catecholamine values were expressed as ng/mg total protein.

### Immunohistochemistry (IHC)

The IHC experiments included two parts. The mice were killed 2 or 6 weeks after the LPS treatment. Mice (n = 6) were deeply anesthetized with a high dose of sodium pentobarbital (160 mg/kg) and transcardially perfused with 0.01 M of PBS and fixed with 4% of paraformaldehyde in 0.01 M of PBS (PFA). Then, brains were removed, postfixed overnight in 4% of PFA and equilibrated sequentially in 20% and 30% sucrose solutions. The fixed brains were cut into 20 μm sections using a microtome (Leica, CM1950, German). All sections were stored in cryoprotectant until staining. The sections were washed in PBS (0.01 M × 3 minutes), blocked with 5% of normal goat serum (1% BSA, 0.1% Triton X in PBS) and incubated overnight at 4℃ with specific primary antibodies: anti‐IBA1 (1:200, rabbit, Proteintech, Cat. No.: 10904‐1‐AP), anti‐CD11b (1:400, rat, Thermo fisher Scientific, Cat. No.: MA5‐17857), anti‐TH (1:400, mouse, Santa Cruz Biotechnology, Cat. No.: sc‐25269), anti‐LC3b (1:400, rabbit, Abcam, Cat. No: ab63817), anti‐pS129 alpha‐Synuclein (1:400, rabbit, Abcam, Cat. No.: ab51253), anti‐HA‐tag (1:100, mouse, Affinity Biosciences, Cat. No.: T0008), anti‐ubiquitin (1:400, mouse, Abcam, Cat. No.: ab7254) or anti‐RFP (1:400, rabbit, Abcam, Cat. No.: ab62341). Then, sections were washed in PBS (0.01 M × 3 minutes) and incubated with a fluorochrome‐conjugated secondary antibody (Alexa Fluor 488 or Alexa Fluor 594, Abcam). Sections were mounted on slides and cover‐slipped with Vectashield (Vector Laboratories, Cat. No.: H‐1000). IHC results were acquired on an Olympus BX43 fluorescence microscope.

### Western blot

OB, SN and striatum tissues were collected 6 weeks after the beginning of the LPS treatment. Protein extraction and Western blot assays were carried out as previously described. Briefly, tissues were harvested and solubilized for 30 minutes at 4°C in a lysis buffer containing 10 mM of Tris‐HCl (pH 8), 2.5 mM of MgCl2, 5 mM of KCl, 1 mM of dithiothreitol, protease inhibitor mixture (1:100), 1 mM of phenylmethylsulfonyl fluoride (PMSF) and 1 mM of sodium orthovanadate (Sigma‐Aldrich). Following centrifugation at 10 000 rpm for 15 minutes, the supernatant containing the cytoplasmic fraction was collected. Protein concentration for each sample was determined by the bicinchoninic acid (BCA) method using the BCA kit (Shanghai Generay Biotech Co., Ltd., Shanghai, China). Then, the protein samples were concentrated and denatured at 95–100°C for 15 minutes. Sixty‐one hundred micrograms of protein extract (in 20 µL) were subjected to 10% of sodium dodecyl sulfate‐polyacrylamide gel electrophoresis and transferred onto 0.2 μm nitrocellulose membranes (Cat.1620177, Bio‐Rad, Shanghai, China). The membrane blots were blocked with 10% of nonfat dry milk for 2 h (Beyotime, Shanghai, China) at room temperature. The membranes were incubated overnight with mouse monoclonal anti‐alpha‐Synuclein (1:800, mouse, Abcam, Cat No.: ab27766) at 4°C. The membranes were then washed and incubated with secondary antibody (anti‐mouse IgG, 1:10000, catalog # G‐21040, Thermo Fisher) for 1 h at room temperature. The intensity of the protein bands was determined using a scanning Western blot imaging system and quantified with the ImageJ software (NIH, Bethesda, MD, USA). β‐actin (1:5000, rabbit, Bioworld Technology, Cat. No.: AP0060) was used as a protein loading control and internal standard. The researcher who performed the image acquisitions and quantifications was blinded to the experimental design.

### Thioflavin S (ThS) staining

The Thioflavin S staining for aggregated proteins was adapted from a previous study (45). After fixation, the brain tissues were cut into sagittal brain sections and stored in a cryoprotectant (C2999. Sigma) until use. Slices were fixed on glass slides and immersed in 0.01 M of PBS for 10 minutes, followed by immersion in 0.05% KMnO_4_/0.01M PBS for 30 minutes. After rinsing with 0.01M PBS (3 minutes × 3), the sections were destained in 0.2% K_2_S_2_O_5_/0.2% oxalic acid/PBS until the brown color disappeared. After washing, the sections were immersed in freshly filtered 0.0125% Thioflavin S/40% EtOH/60% PBS for 150 sec in the dark. The sections were then incubated in 50% EtOH/PBS for 30 minutes. Sections were coverslipped prior to imaging.

### Stereotaxic injection of AAV‐mutant‐α‐Syn

Our previous studies showed that the mutant α‐Syn could transmit beyond the synapses to regions beyond the sites of AAV infected cells (41). To determine if the IL‐1R1 signaling affects the transsynaptic transmission of mutant α‐Syn from the OB, the AAV1/2 expressing mutant α‐Syn‐GFP (41) was injected into the OB of the IL‐1R1^GR/GR^ and IL‐1R1^r/r^ mice (30). Two weeks after the AAV injection, the mice were sacrificed and perfused with 4% of PFA in 0.01 M of PBS. Striatum region was sampled to detect the mutant α‐Syn‐GFP.

### Statistics

All data were mean ± SD (standard deviation) and analyzed using the SPSS16.0. Differences between means of normally distributed data were analyzed by two‐tailed Student’s *t* test when comparing two groups or one‐way ANOVA with (Fisher's least significant difference) LSD multiple comparison *post hoc* tests when comparing more than two groups. For non‐normally distributed data, a nonparametric two‐tailed Mann–Whitney U test was used when comparing two groups or a Kruskal–Wallis test with Dunn’s multiple comparison *post hoc* tests when assessing more than two groups. *P* < 0.05 was considered significant.

## Results

### Chronic LPS intranasal infusion activates microglia in the OB and SN

**Figure 1:**
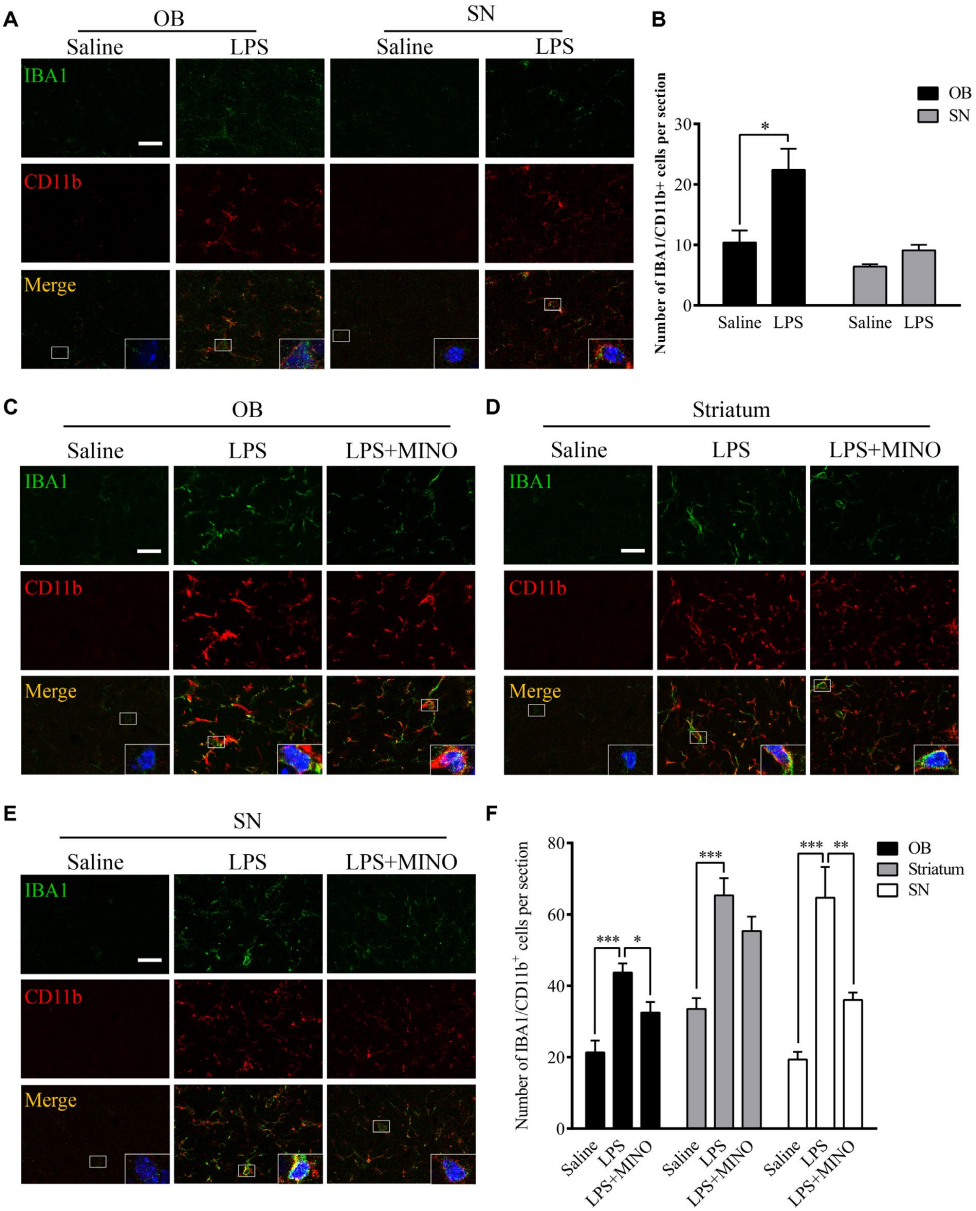
Effects of chronic LPS intranasal infusion on microglia activation in the olfactory bulb in wild‐type mice. **A.** Representative images of IBA1/CD11b^+^ staining in the OB and SN 2 weeks after the LPS treatment. **B.** Quantitative analysis of the IBA1/CD11b^+^ cell in the OB and SN 2 weeks after the LPS treatment (n = 3). **C**‐**E**. Representative images of IBA1/CD11b^+^ staining in the OB, striatum and SN 6 weeks after the LPS treatment. **F**. Quantitative comparison of IBA1/CD11b^+^ cell numbers in the OB, striatum and SN 6 weeks after the LPS treatment (n = 3). One‐way ANOVA analysis followed by LSD multiple comparison was used to contrast the groups’ difference. *P < 0.05, **P < 0.01, ***P < 0.001. Scale bar = 20 μm.

Inflammatory responses in the olfactory system can be induced through exposure of environmental toxins in the nasal cavity. Anatomical studies indicated that olfactory sensory neurons located in the olfactory epithelium extend their axons solely to the olfactory bulb (40). In this study, LPS was intranasally delivered to mice as an environmental toxin to induce inflammation in OB. The results showed that the LPS treatment for two consecutive weeks increased IBA1 intensity in the OB, not SN. In addition, the number of IBA1/CD11b^+^ cells in OB, not SN, was increased in the LPS‐treated group in comparison to the control group (Figure 1A,B, *P* < 0.05). Further, continuous LPS treatment for six consecutive weeks increased IBA1 intensity in the OB, SN and striatum in comparison to saline‐treated mice (Figure 1C‐F). Treatment with minocycline (MINO), a microglial inhibitor, reduced microglial numbers in the OB (*P* = 0.018), SN (*P* = 0.009) and striatum (*P* = 0.143) of the LPS‐treated mice. These results show microglia activation induced by the LPS treatment initially occurred in the OB, but reached the SN later.

### Chronic LPS intranasal infusion‐induced IL‐1β expression and α‐Syn overexpression and aggregation in the OB in an IL‐1β/IL‐1R1‐dependent manner

**Figure 2:**
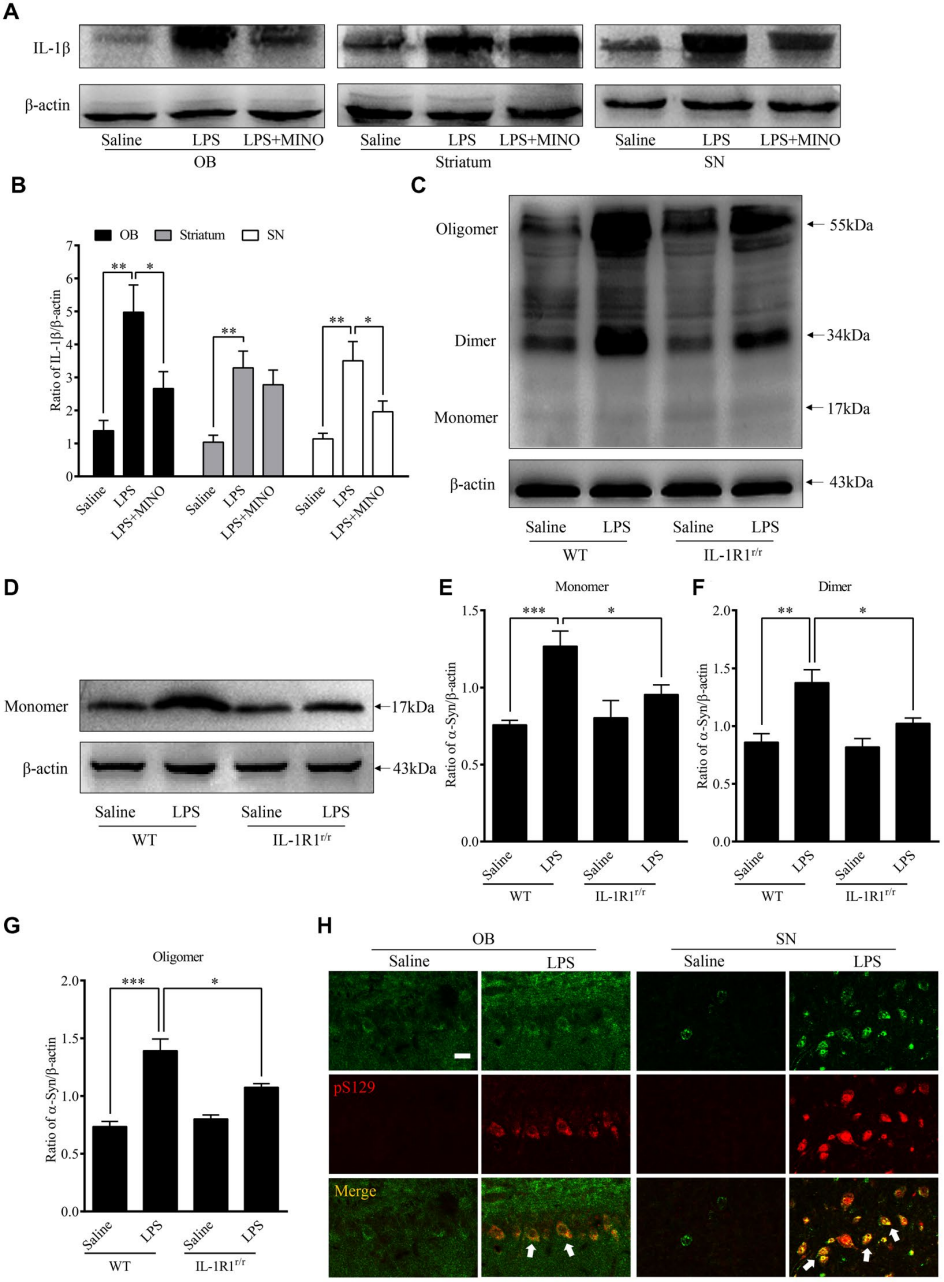
Activated microglia‐induced α‐Syn overexpression and aggregation in the OB. **A.** Representative Western immunoblot images of IL‐1β from the OB, striatum and SN of animals treated with LPS or saline infusion. **B.** Comparison of normalized IL‐1β expression levels in the OB, striatum and SN. **C.** Representative Western blot images of α‐Syn aggregation in the OB of WT (GR/GR) and IL‐1R1^r/r^ mice. **D**. Representative Western blot image of α‐Syn (17 kDa) in the OB of WT (GR/GR) and IL‐1R1^r/r^ mice. **E**. Comparison of normalized fibrillar α‐Syn aggregation levels (55 kDa) in the OB of WT and IL‐1R1^r/r^ mice. n = 6; Comparison of normalized oligomeric α‐Syn aggregation levels (34 kDa) in the OB of WT and IL‐1R1^r/r^ mice. n = 6; **G.** Comparison of normalized monomeric α‐Syn aggregation levels (17 kDa) in the OB of WT and IL‐1R1^r/r^ mice. n = 6. **H.** Representative images of IL‐1R1 (HA) and pS129 α‐Syn positive staining in the OB and SN. β‐actin was used as a loading control. One‐way ANOVA analysis followed by LSD multiple comparison was used to contrast the groups’ difference. n = 6 *P < 0.05, **P < 0.01, ***P < 0.001.

Previous studies have shown that activated microglia release inflammatory cytokines, including IL‐1β (53). In this study, the levels of IL‐1β were quantified (Figure 2A,B). LPS treatment (for 6 weeks) increased IL‐1β levels in the OB (*P* = 0.002), SN (*P* = 0.003) and striatum (*P* = 0.002) in comparison to those treated with saline. In addition, MINO pretreatment attenuated the LPS‐induced increases of IL‐1β in the OB (*P* = 0.038), SN (*P* = 0.043), but not striatum (*P* = 0.466). The results suggest that the enhanced IL‐1β in the OB, and SN was released from activated microglial cells.

Western blot analysis was used to measure α‐Syn levels and to determine the effects of microglial activation on α‐Syn levels in the OB 6 weeks after the LPS treatment. Figure 2 shows detection of α‐Syn in the OB following the LPS treatment (Figure 2C‐G): the LPS treatment caused increased α‐Syn levels (17‐kd size band monomeric α‐Syn, *P* < 0.05) and increased aggregation of α‐Syn in the OB including the oligomeric (34‐kd size band, *P* < 0.01) and fibrillar alpha‐Synuclein (54‐kd size band, *P* < 0.001).

In addition, we found that the level of the monomeric α‐Syn in the OB of IL‐1R1^r/r^ (IL‐1R1 knockout) mice was significantly lower than that in WT mice (*P* = 0.038, Figure 2D) after the LPS treatment. Importantly, the aggregative α‐Syn (oligomer, *P* = 0.031 and fibrillar, *P* = 0.022) levels were also decreased in the OB of IL‐1R1^r/r^ mice following the LPS treatment. Therefore, chronic LPS intranasal infusion‐induced α‐Syn overexpression and aggregation in the OB is IL‐1R1 dependent.

### Chronic LPS intranasal infusion‐induced phosphorylated α‐Syn in the OB of mice

**Figure 3:**
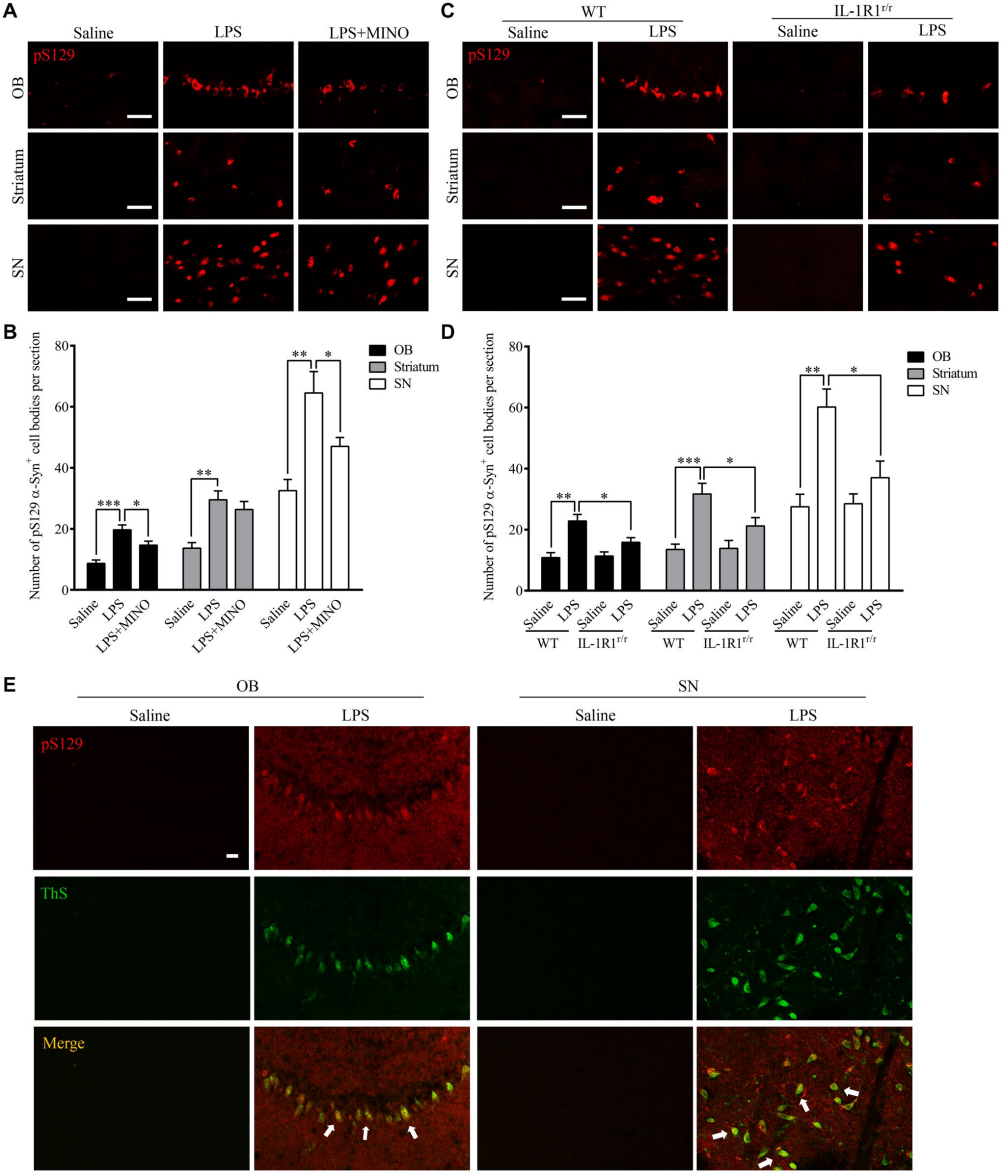
Chronic LPS intranasal infusion‐induced phosphorylation of S129 α‐Syn in the OB, SN and striatum. **A.** Representative images of immunohistochemical staining for pS129 α‐Syn in the OB, SN and striatum of mice following LPS intranasal infusion with/without MINO inhibition of microglia. **B.** Quantitative analysis of pS129 α‐Syn in the OB, SN and striatum (n = 3). **C.** Representative images of immunohistochemical staining for pS129 α‐Syn in the OB, SN and striatum following LPS intranasal infusion in WT or IL‐1R1^r/r^ mice. **D**. Quantitative analysis of pS129 α‐Syn in the OB, SN and striatum reported in **C**. **E**. n = 3. Double‐immunostaining of pS129 α‐Syn (red) and ThS (green) in the OB of LPS‐treated WT mice. One‐way ANOVA analysis followed by LSD multiple comparison was used to contrast the groups’ difference. *P < 0.05, **P < 0.01, ***P < 0.001. [Colour figure can be viewed at wileyonlinelibrary.com]

During PD progression, α‐Syn pathological lesions include extensively phosphorylated α‐Syn at serine 129 (pS129 α‐Syn), which plays a critical role in synucleinopathy. The phosphorylation of α‐Syn at Ser129 regulates α‐Syn fibril formation, which leads to enhanced α‐Syn toxicity (50). pS129 α‐Syn is, therefore, a marker for a‐Syn aggregation and cytotoxicity. To examine whether native α‐Syn is thus phosphorylated after the LPS treatment, pS129 α‐Syn was labeled by IHC. The results show increased pS129 α‐Syn positive cells in the OB (*P* < 0.001), SN (*P* = 0.002) and striatum (*P* = 0.001) of the LPS‐treated mice than those in saline‐treated mice (three sections per brain region of every mouse) (Figure 3A,B). Moreover, inhibiting microglia activity with MINO reduced the number of pS129 α‐Syn positive cells in the LPS‐treated mice, suggesting that microglial activation played a vital role in the phosphorylation of α‐Syn at S129. In addition, in the IL‐1R1r/r mice, less pS129 α‐Syn positive cells were found in the OB (*P* < 0.001), SN (*P* = 0.002) and striatum (*P* = 0.001) of the LPS‐treated mice (three sections per brain region of every mouse) (Figure 3C,D). This result suggests that the LPS‐induced phosphorylation of α‐Syn at S129 is IL‐1R1 dependent. To confirm that the presence of pS129 α‐Syn reflects the aggregation of α‐Syn, pS129 α‐Syn was double‐labeled with ThS, results show this marker of protein aggregation is co‐localized with pS129 α‐Syn (Figure 3E). Thus, chronic LPS intranasal infusion induces phosphorylated α‐Syn and protein aggregation in the OB, SN and striatum. We also used the IL‐1R1 reporter mice (IL‐1R1^GR/GR^) which allows detection of IL‐1R1 protein by the tracking epitope HA. The IHC staining showed that IL‐1R1 in OB and SN (Figure 2H) was co‐localized in cells with pS129 α‐Syn.

### Deletion of IL‐1R1 inhibited the transmission of α‐Syn from the OB to SN

**Figure 4:**
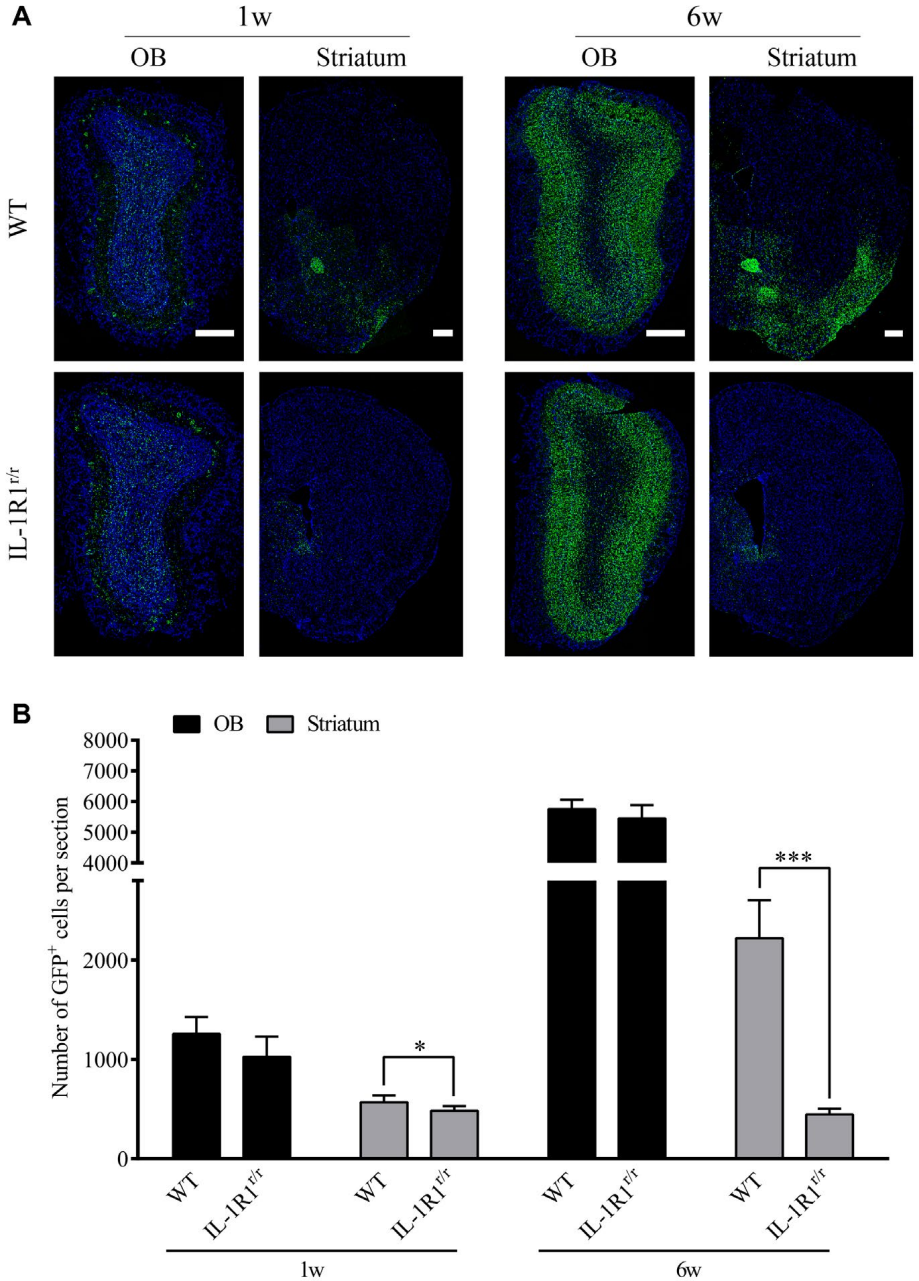
Knockout of IL‐1 receptor‐1 affects the transmission of α‐Syn‐GFP from the OB to SN. **A.** Representative images of AAV‐mutant α‐Syn‐GFP expression in WT (n = 5) and IL‐1R1^r/r^ mice (n = 5). Scale bar = 200 μm. **B.** Quantitative analysis of α‐Syn‐GFP positive cell number in the OB and striatum of WT and IL‐1R1^r/r^ mice. Independence‐sample student test was used in the statistical analysis. *P < 0.05.***P < 0.001.

Previous studies have found that mutant α‐Syn expressed in the OB by an associated adenovirus (AAV) vector can be transferred to sensitive brain regions beyond the immediate synaptic connections of the OB and contributes to dopaminergic cell loss in the SN (41). Given that IL‐1β/IL‐1R1 signaling was found to affect the production of α‐Syn aggregates in areas beyond the OB in this study, an adeno‐associated adenovirus (AAV) vector expressing the mutant α‐Syn‐GFP fusion proteins was injected to IL‐1R1^r/r^ mice to determine if the lack of IL‐1R1 affected the transmission of the pathological α‐Syn. The results show that mutant α‐Syn‐GFP was expressed in the OB of WT and IL‐1R1^r/r^ (WT vs. IL‐1R1^r/r^, *P* = 0.332) mice 1 week after the AAV injection, indicating that the mutant α‐Syn‐GFP was similarly expressed in both mice (Figure 4A&B). In contrast, significantly more α‐Syn‐GFP was found in the striatum of WT mice than that in the IL‐1R1^r/r^ mice (WT vs. IL‐1R1^r/r^, *P* = 0.034) 6 weeks after AAV injection. These data indicate that the transfer of α‐Syn‐GFP beyond the OB is facilitated by IL‐1R1.

### Chronic intranasal LPS infusion‐induced TH‐positive cell loss in the nigral‐striatal system

**Figure 5:**
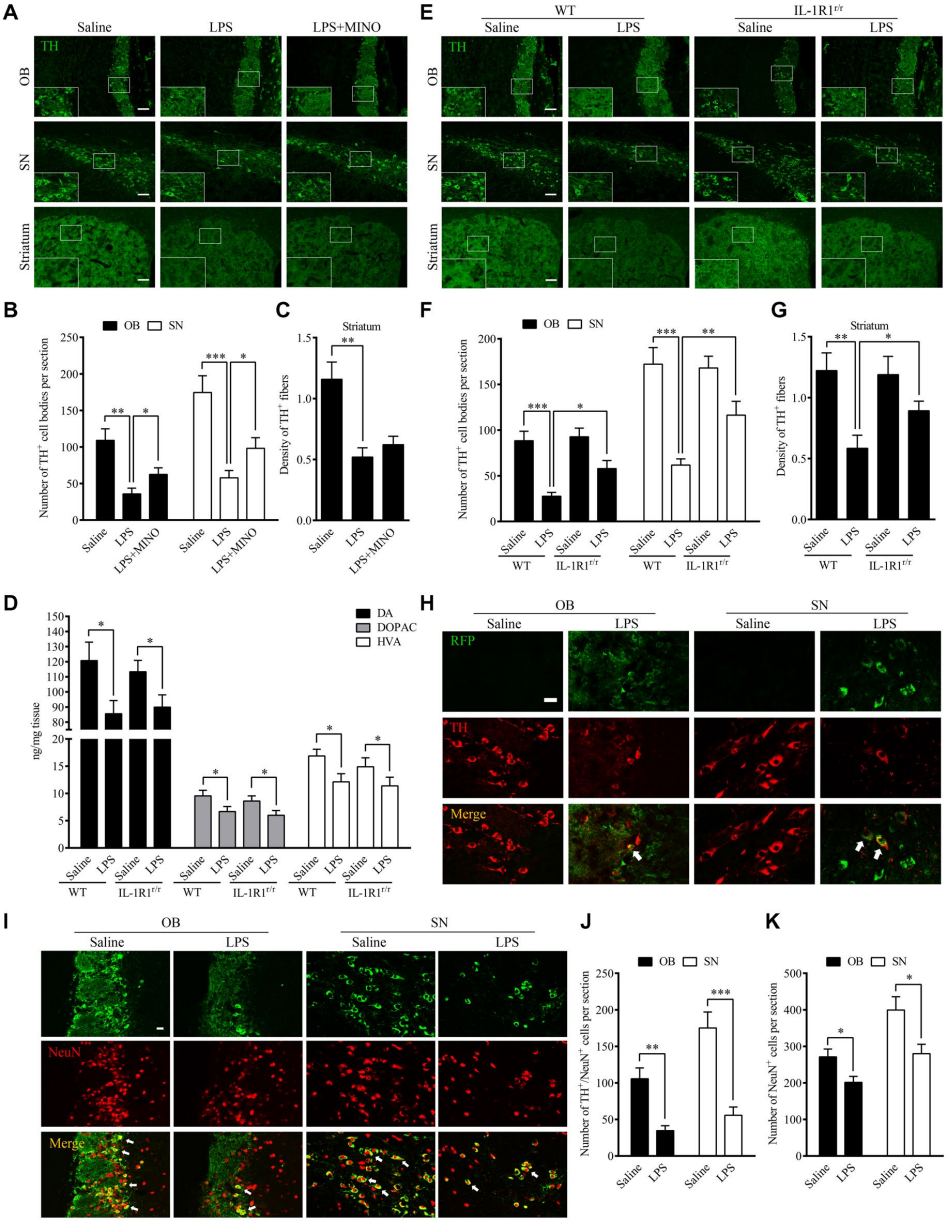
Chronic intranasal LPS infusion‐induced TH‐positive cell loss in the nigral‐striatal regions. **A.** Representative images of tyrosine‐hydroxylase positive staining in the OB, SN and striatum of WT mice following 6 weeks of intranasal LPS infusion. Scale bar = 40 μm. **B.** Quantitative analysis of TH‐positive cells in the OB and SN of mice (n = 3). **C.** Quantitative analysis of TH‐positive fiber in the striatum of mice. **D**. Quantities of dopamine and its metabolites, as measured by HPLC, in the striatum of WT mice following saline or LPS intranasal infusion. **E**. Representative images of TH immunohistochemical staining in the OB, SN and striatum following LPS intranasal infusion in WT and IL‐1R1^r/r^ mice (n = 3). Scale bar = 40 μm. **F**. Quantitative analysis of TH‐positive cells in the OB and SN of WT and IL‐1R1^r/r^ mice (n = 3). **G**. Quantitative analysis of TH‐positive fiber in the striatum of IL‐1R1^r/r^ mice. **H.** Double‐immunostaining of TH (red) and RFP, recognizing IL‐1R1 (green) in the OB and SN of IL‐1R1^GR/GR^ mice. **I.** Representative images of TH/NeuN cells in OB and SN. **J** and **K.** Quantitative analysis of TH^+^/NeuN^+^ cells and NeuN^+^ cells in the OB and SN. For contrasting between two groups, the independence‐sample student test was used in the experiment. And for contrasting among more than two groups, one‐way ANOVA analysis followed by LSD multiple comparison was used to contrast the groups’ difference. *P < 0.05, **P < 0.01, ***P < 0.001. Scale bar = 10 μm.

To determine the impact of chronic LPS intranasal infusion on dopaminergic cell loss in the nigral‐striatal system, the number of tyrosine hydroxylase (TH) immuno‐reactive cells in the OB and SN were stained 6 weeks after the LPS treatment. The density of TH‐positive nerve fibers was also determined in the striatum. The results show that the number of tyrosine hydroxylase (TH) immuno‐reactive cells was reduced in the OB (*P* = 0.002) and SN regions (*P* < 0.001) after 6 weeks of the LPS treatment, indicating loss of dopaminergic cells (Figure 5A,B). Moreover, the density of TH‐positive nerve fibers in the striatum was significantly reduced after 6 weeks of the LPS treatment (*P* = 0.003, Figure 5C). This indicates neural terminals of dopaminergic cells were decreased in the striatum. In addition, HPLC analysis found that mice after the LPS treatment had reduced striatal levels of DA (*P* = 0.001), DOPAC (*P* = 0.002) and HVA (*P* = 0.002) in comparison with saline‐infused control mice (Figure 5D). Notably, IL‐1R1^r/r^ mice (Figure E‐G) did not display significant difference after the LPS treatment. These data demonstrate that the LPS treatment caused IL‐1R1‐dependent impairment of nigral‐striatal dopaminergic system.

To determine whether IL‐1R1 is located on dopaminergic cell bodies in the OB and SN of the IL‐1R1 reporter (IL‐1R1^GR/GR^) mice, double label immunofluorescence was performed. The results (Figure 5H) show that IL‐1R1 (RFP, the knockin tracer of IL‐1R1 mRNA in the reporter mice) was expressed on dopaminergic cell bodies (TH‐positive signals), suggesting there may be direct intracellular signaling of IL‐1R1 on these neurons that contributes to the dopaminergic cell loss. Whether the α‐Syn or its aggregates interact with IL‐1R1 or the transmission of α‐Syn‐GFP is promoted by the dopaminergic IL‐1R1 remains to be determined.

To verify that the loss of TH‐positive cells reflected a loss of dopaminergic neurons, not simply a loss of TH expression, we analyzed OB and SN sections stained with a neuronal marker NeuN. Results show double‐labeled TH+/NeuN + cells and single‐labeled NeuN + cells in Figure 5I‐K. The numbers of the TH+/NeuN + cells in OB (vs. control, *P* < 0.05) and SN (vs. control, *P* < 0.05) were reduced in the LPS treatment group. In parallel, the numbers of NeuN + cells in OB (vs. control, *P* < 0.05) and SN (vs. control, *P* < 0.05) were also reduced by the LPS treatment. Thus, the LPS treatment resulted in the loss of TH + cells in the OB and SN.

### Chronic intranasal LPS infusion‐induced motor‐related behavioral dysfunction

**Figure 6:**
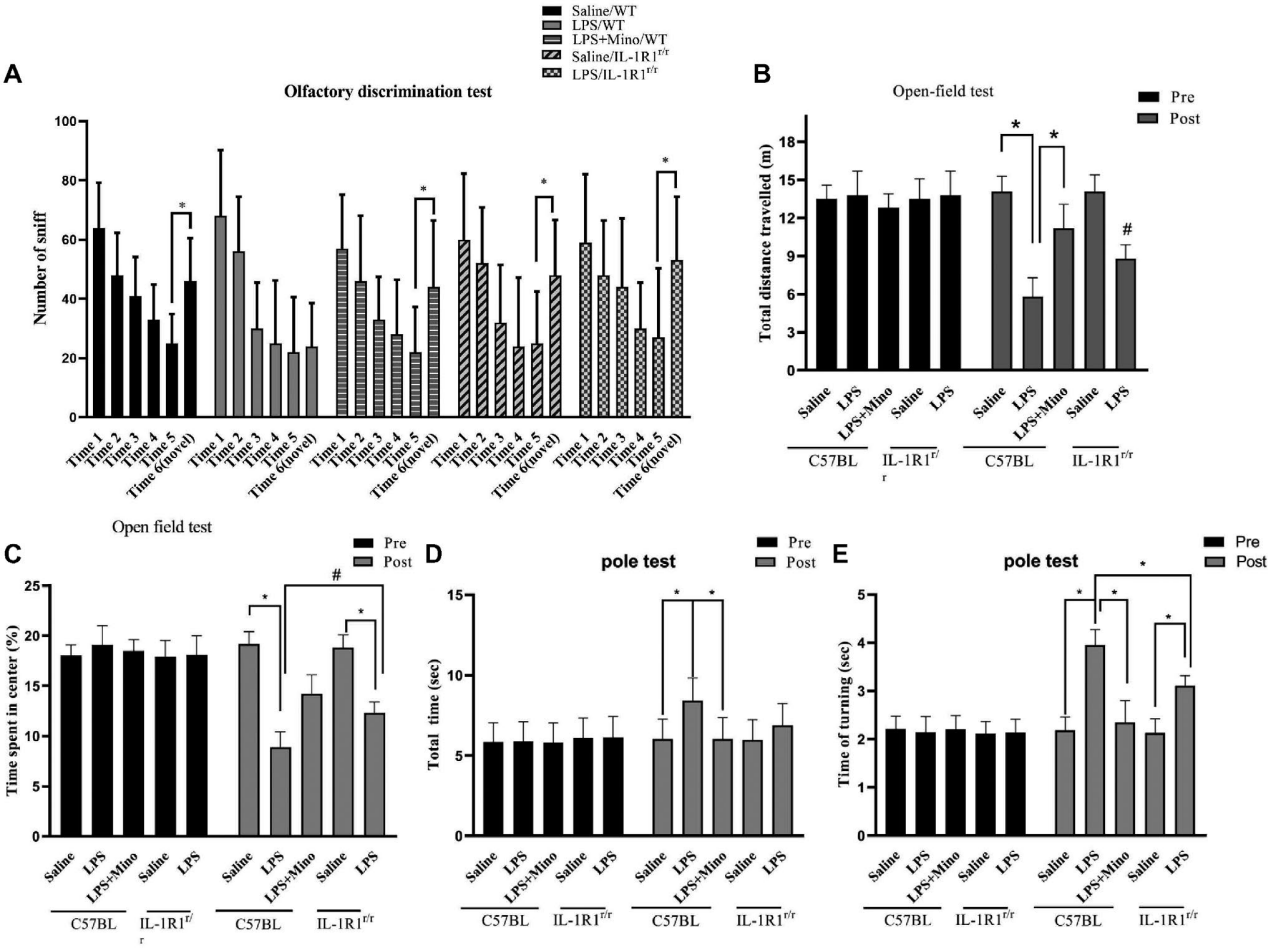
The effects of chronic intranasal LPS infusion on motor‐related behavioral activities in WT and IL‐1R1^r/r^ mice. **A**. Olfactory discrimination test. **B** and **C.** Locomotor behavior (total distance traveled and time spent in the center of the field) in the open‐field test of saline effused and LPS mice. D&E) Total time and time turning in the pole test of saline effused and LPS mice. The data of each group per odor test was analyzed using two‐way ANOVA followed by Newman–Keuls or a similar post hoc test to determine significant habituation (less time sniffing successive same smells), and dishabitutation (more time sniffing a novel smell). One‐way ANOVA analysis followed by LSD multiple comparison was used to contrast the groups’ difference for the open‐field and pole test data in the experiment. *P < 0.05, ^#^ *P* < 0.05.

Previous studies have shown that overexpression of the mutant α‐Syn in the OB‐induced motor and non‐motor PD‐like symptoms (41). It is unknown whether the α‐Syn pathology caused by our LPS treatment would also induce PD‐like motor and non‐motor dysfunction. To answer this question, a series of behavioral tests was performed. In the olfactory discrimination test, the data indicate that the LPS treatment resulted in reduced olfactory discrimination (Figure 6A) in WT mice (saline/WT vs. LPS/WT, p < 0.05). In addition, inhibition of microglial activation by Mino reversed this effect of the LPS treatment (Figure 6C). Further, the impaired ability of olfactory discrimination by LPS treatment was attenuated in the IL‐1R1r/r mice (*P* < 0.05, Figure 6A).

In the open‐field test, we found that the total distance traveled and time spent in center were lower in the LPS‐treated WT mice (distance: *P* < 0.001, time in center, *P* < 0.05, Figure 6B,C). Similarly, treatment with MINO reversed these effects. Furthermore, the LPS‐induced reduction in locomotor activity was again attenuated in the IL‐1R1r/r mice (*P* < 0.05). Additionally, the LPS‐treated induced bradykinesia in the WT, but not in the IL‐1R1^r/r^, mice in the pole test (Figure 6D,E).

### Chronic intranasal LPS infusion differentially regulated autophagy in the OB and SN

**Figure 7:**
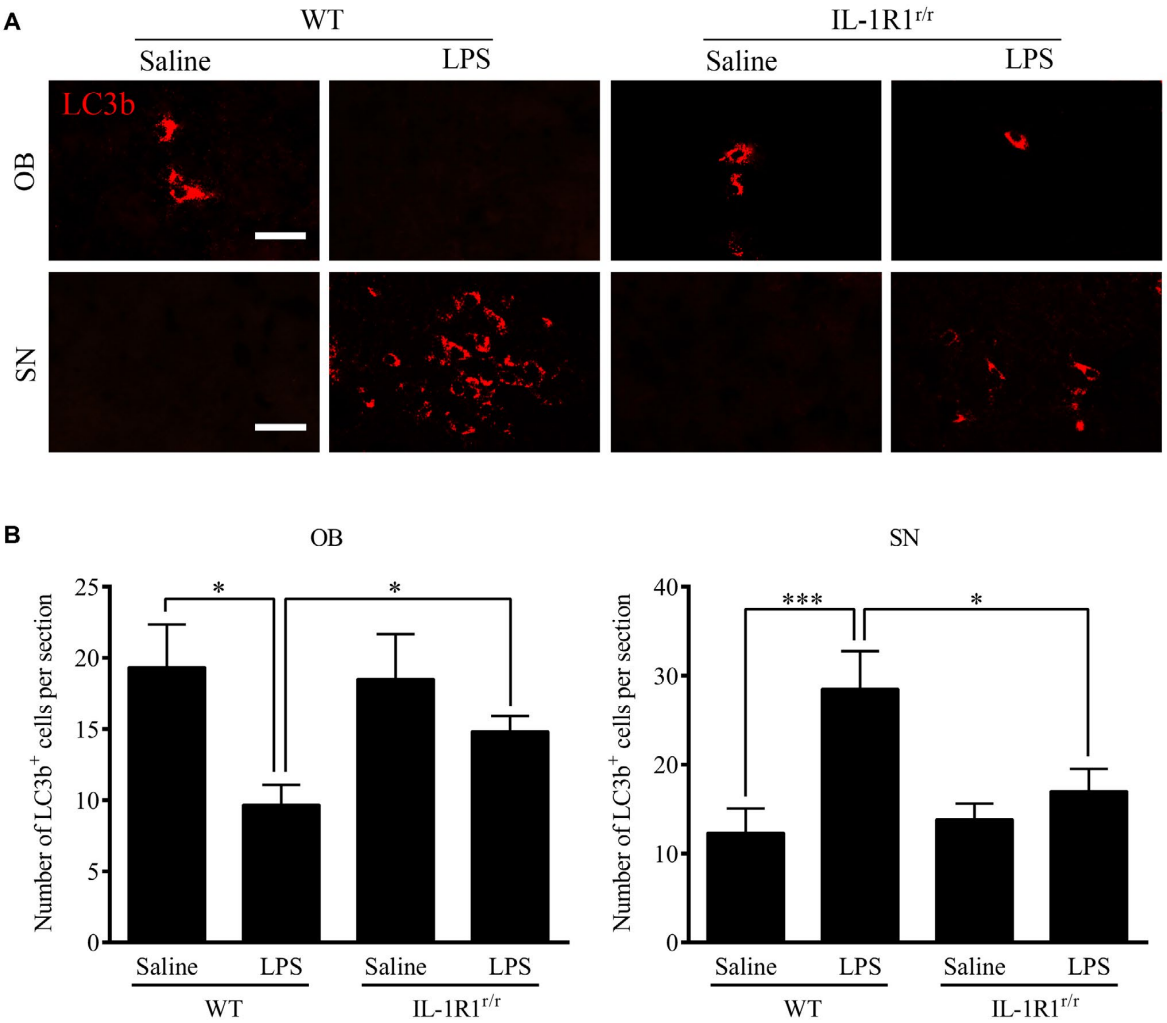
Activated microglia induced by chronic intranasal LPS infusion increased autophagic pathways. **A.** Representative images of immunohistochemical staining of LC3b in the OB & SN of WT and IL‐1R1^r/r^ mice. **B.** Quantitative analysis of LC3b in the OB and SN of WT and IL‐1R1^r/r^ mice (n = 3). One‐way ANOVA analysis followed by LSD multiple comparison was used to contrast the groups’ difference. *P < 0.05, ***P < 0.001, Scale bar = 20 μm.

Previous studies have demonstrated that IL‐1R1 activation regulates the basal activity of autophagy. As autophagy is important for the clearance of aggregated misfolded proteins (17), we analyzed whether LPS‐induced elevation of IL‐1β/IL‐1R1 signaling affected autophagy and the relationship between autophagy and the increased α‐Syn levels. The microtubule‐associated protein LC3b was used as an autophagy marker. LC3b is a constituent of the autolysosome membrane and is eventually degraded by lysosomal hydrolases. The results show that the number of LC3b‐positive cells in the OB of the LPS‐treated WT mice was reduced in comparison to the saline‐treated control mice (Figure 7A,B, *P* = 0.009). The number of LC3b‐positive cells in the OB in the LPS‐treated IL‐1R1^r/r^ mice was not different in comparison to the saline‐treated IL‐1R1^r/r^ mice. On the contrary, the number of LC3b‐positive cells in the SN (*P* = 0.021) was increased the LPS‐treated WT mice in comparison to the saline‐treated control mice. Again, this effect is absent in IL‐1R1^r/r^ mice.

### LPS could not enter the brain to activate the microglia

**Figure 8:**
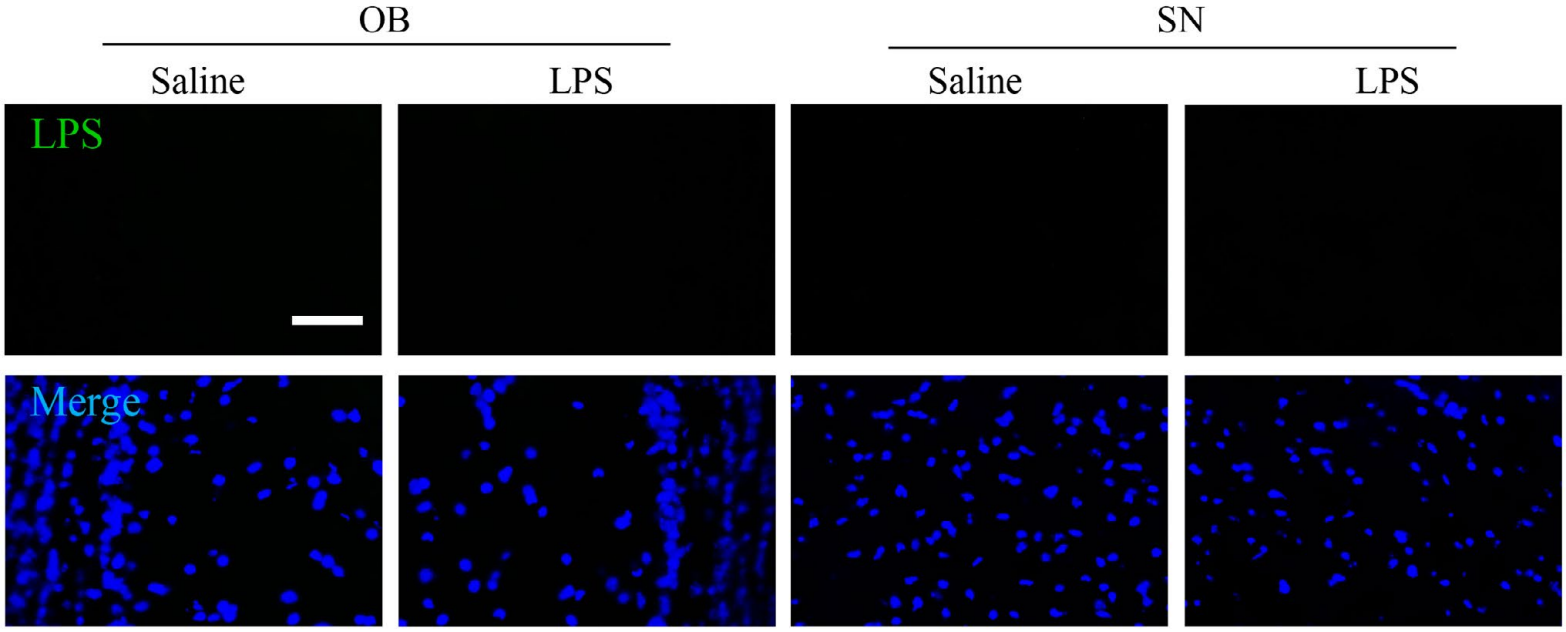
Effect of LPS treatment over 6 weeks on the uptake of LPS by brain. Results shows that LPS treatments does not increase LPS uptake by brain.

One mechanistic consideration is whether the intranasally administered LPS can leak into the brain. Because OB is anatomically connected to this site of the LPS administration, it is important to determine whether LPS could enter the brain to trigger OB inflammation. We show here that LPS was not detected in the OB, and SN field by the immunohistochemistry (Figure 8). This result is consistent with previous studies that most effects of peripherally administered LPS is mediated through LPS receptors located outside the blood–brain barrier (BBB). Thus, the observed OB microglial activation is not caused by the entry of LPS into the brain parenchyma, but by indirect pathways, probably involving activation of the cells of the blood–brain barrier.

## Discussion

Mounting evidence indicates that neuroinflammation contributes to the development of PD and other degenerative diseases (55). In the central nervous system, the olfaction sensory neurons are particularly susceptible to neuroinflammatory attack due to its accessibility to inhaled inflammagens in the environment. Thus, beyond detecting environmental hazards, pheromones and food, the olfaction system is often affected by environmental toxins leading to immune responses (9). In the current literature, an important theory for the initiation and progression of several neurodegenerative diseases involves neuroinflammation initiated from the OB, and then, propagated to other brain regions. Indeed, olfactory dysfunction has been considered as a vital pathogenic factor in early onset of PD (14). This is supported by clinical observations that olfactory dysfunction and Lewy body deposition in olfactory‐related fields occur in the early stages of PD. The present study is designed to determine the role of IL‐1, a critical neuroinflammatory cytokine, in mediating the initiation and propagation of neuroinflammation and pathogenic α‐Syn from the OB in a model of olfactory inflammation‐induced PD‐like neuropathology. We found intranasal lipopolysaccharide infusion activated microglia in the OB, SN and striatum. In association, α‐Syn is overexpressed in the OB in an IL‐1R1‐dependent manner. In addition, activated microglia is associated with reduced autophagy in the OB, further contributing to α‐Syn aggregation. IL‐1R1 is also found to be critical for the transmission of pathogenic α‐Syn to the SN from OB, leading to enhance the autophagy in the SN, neurotoxicity and impaired function of the dopaminergic system in the SN and striatum, and ultimately motor dysfunction.

The olfactory system is continuously exposed to a variety of potentially harmful environmental agents, such as bacteria, viruses, mold, dust, pollen and environmental chemicals. These environmental toxins can easily cause inflammation in the olfactory mucosa (OM) and activate microglia in the olfactory bulb (48). Previous studies found mice treated with the intranasally instilled PR8 influenza virus show inflammation in the OB and lung. The inflammation lasted 15 h in the lung, but 96 h in the OB (18, 33), indicating the olfactory system is a particularly vulnerable to environmental toxins‐induced inflammation (21, 42). The naris of mice was exposed to LPS to induce inflammation in the OB and the brain (39, 42). Although, it was found that there were sex differences and the rating of mortality following induction of olfactory α‐Synucleinopathy (36). The inflammation of olfactory system plays a necessary during the beginning of α‐Synucleinopathy. Our data show that LPS is a macromolecular that does not enter the brain across the blood–brain barrier. The sequential inflammatory activation of the OM and subsequent expression of inflammatory cytokines within the brain parenchyma is likely a result of the propagation of inflammation (22, 51). Previous studies found activated microglia are the major source of brain inflammatory cytokines after peripheral systemic LPS challenge (4). Consistent with this, we found the number of IBA1/CD11b + cells and levels of IL‐1β were increased following the LPS treatment, suggesting the LPS activated microglia drive intra‐parenchyma neuroinflammation. Minocycline had been used to prevent microglia activation and cytokine production in the past (1). Our results show minocycline is also effective in reducing the LPS treatment‐induced microglia activation and the induction of pro‐inflammatory cytokine expression. Thus, intranasal LPS activated microglia along the olfactory pathway, leading to neuroinflammation in the OB.

Recent studies have suggested that PD pathogenesis is divided into three temporal phases/factors: triggers, facilitators and aggravators (23). During the first phase, viral infections or environmental toxins trigger the inflammatory process in the brain and/or peripheral tissues.(44) The inflammation then induce microenvironmental changes in the brain, leading to a primed condition for the development of PD pathology (15). The induction of microglial activation could result in this condition. However, it must be pointed out that microglia activation alone cannot induce PD pathology. To do so, inflammatory signals need to reach the relevant neurons. We show in this study that α‐Syn and IL‐1R1 are co‐localized in the mitral cell layer of the OB. Therefore, IL‐1β produced by microglia could act on these IL‐1R1 expressing neurons to cause neuropathology. Indeed, α‐Syn overexpressing cells are co‐localized with IL‐1R1. In addition, α‐Syn levels including the monomers, oligomers and fibril in OB were elevated following the LPS treatment in an IL‐1R1‐dependent manner. Further, the ps129‐α‐Syn, a marker for the aggregative α‐Syn, was induced in OB, SN and striatum following the LPS treatment. The aggregative α‐Syn was confirmed by the immunohistochemical labeling of ThS. Importantly, mice treated with the LPS showed the loss of TH‐positive cells in SN, decreased dopamine and its metabolites in striatum and PD‐like behaviors as those observed previously in other PD models (35). Because the intranasal LPS treatment in IL‐1R1^r/r^ mice did not increase the expression of α‐Syn and all the downstream consequences, the present results demonstrate for the first time that IL‐1R1 was necessary for the intranasal LPS‐induced α‐Syn overexpression in the mitral cell layer and in the striatum‐SN system.

Overexpression of α‐Syn‐GFP fusion protein in the OB was found to induce the prodromal symptoms of PD in previous studies conducted on rats (29, 41). In this study, an α‐Syn‐GFP AAV was used to overexpress the mutant α‐Syn in the OB to determine whether the overexpressed mutant α‐Syn would induce PD pathology in IL‐1R1 deficient (IL‐1R1^r/r^) mice. Results show that mutant α‐Syn overexpressed in the OB of IL‐1R1^r/r^ mice and WT mice at similar levels. In contrast, significantly less alpha‐Syn‐GFP fusion protein was detected in the striatum in the IL‐1R1^r/r^ mice than in the control mice. It should be noted that it is known that proteins expressed by the AAV does not transmit beyond one neuronal cell (41) from the infected cell and AAV itself does not migrate to distant brain regions (our unpublished data). Therefore, our result indicates that transference of the pathological α‐Syn to the SN was reduced without IL‐1R1. Thus, IL‐1R1 is pivotal for the transfer of pathogenic α‐Syn from OB to SN. Interestingly, our data showed that the α‐Syn‐GFP was also detected in the anterior commissure of WT, but not IL‐1R1^r/r^, mice. Because no neuronal IL‐1R1 was not found in this region, the mechanism for this phenomenon remains unclear.

We show here that intranasal LPS treatment activates microglia to release IL‐1β. Although previous studies speculated that IL‐1β signaling is important for the pathogenesis of PD, the mechanism of how IL‐1 might affect relevant neurons is not known. The results of the present study show IL‐1R1 is co‐localize with p‐α‐Syn expressed in the mitral cell layer, suggesting the neuroinflammation driven by microglial IL‐1β may find its target on neuronal IL‐1R1 to trigger α‐Syn pathology.

Previous studies found that the ubiquitin‐proteasome system (UPS) and the autophagy‐lysosomal pathway (ALP) were the two major cellular mechanisms responsible for clearance of proteins (56). The former degrades short‐lived soluble proteins, the latter is the response for degradation of long‐lived protein aggregates to maintain cellular homeostasis in differentiated, postmitotic neuronal cells. Aggregated α‐Syn is also known to be metabolized by various lysosomal enzymes by the autophagy processes in the mitral cell (7). In periphery, it is well established that Th1 cytokines, including IFN‐γ, TNF‐α, IL‐1, IL‐2, IL‐6 and TGF‐β, induce the effects of autophagy, while classical Th2 cytokines including IL‐4, IL‐10 and IL‐13, inhibit the autophagy effects (31, 60). We therefore investigated the role of IL‐1R1 on autophagy of neurons after the LPS treatment. Our data show that the autophagic marker LC3b was reduced by the LPS treatment in wild‐type mice in the OB, but this reduction is attenuated in the IL‐1R1^r/r^ mice. This could suggest that IL‐1R1 in the OB plays a role in the LPS‐induced inhibition of autophagy, consistent with the role of IL‐1R1 signaling in contributing to the impaired clearance of α‐Syn in the OB. Paradoxically, the autophagy in the SN appears to be enhanced. This might be explained by the fact that pathological and aggregated α‐Syn is transmitted from the OB to SN, which induce the autophagic reactions to clear it.

In conclusion, environmental toxins inducing olfactory inflammation contribute to α‐Synuclein pathology in an IL‐1β/IL‐1R1‐dependent manner. The IL‐1β/IL‐1R1 pathway plays a vital role as a trigger, facilitator and aggravator in the three temporal phases of PD pathology and should be considered a potential therapeutic target against PD progression.

## Conflict of Interest

None.

## Data Availability

Data are available upon request to the corresponding author.
